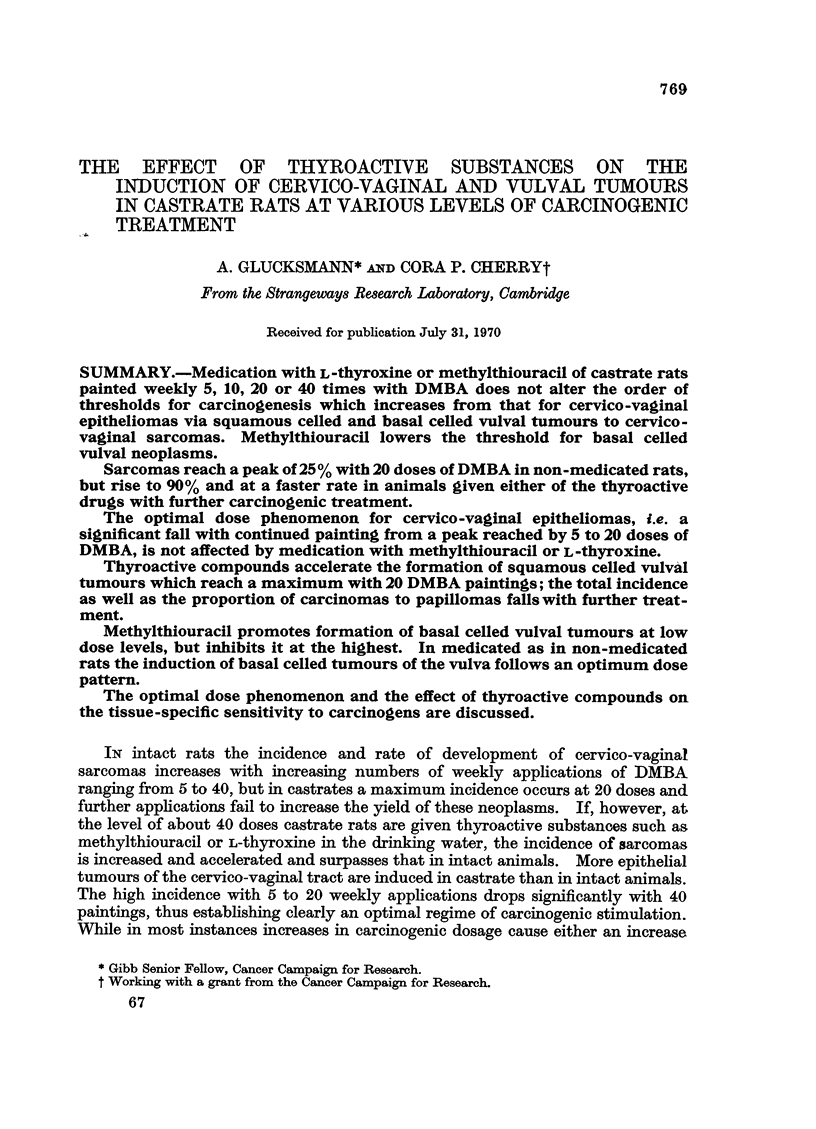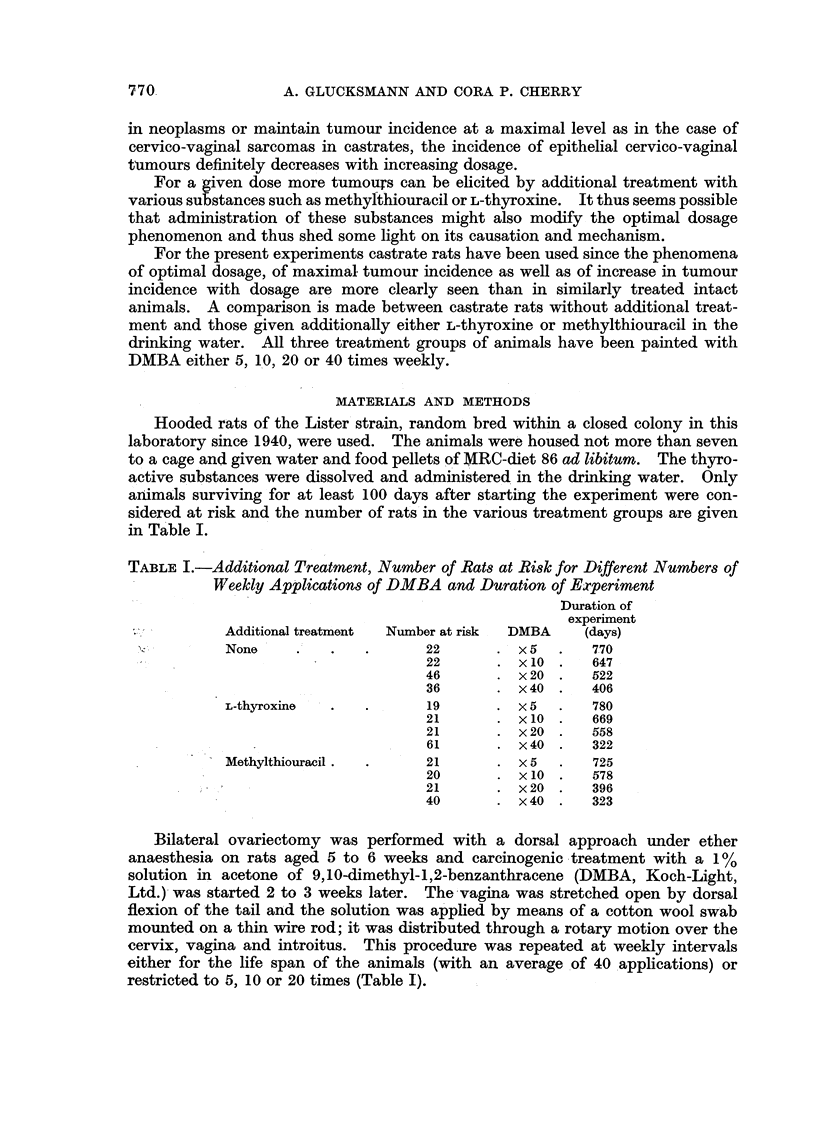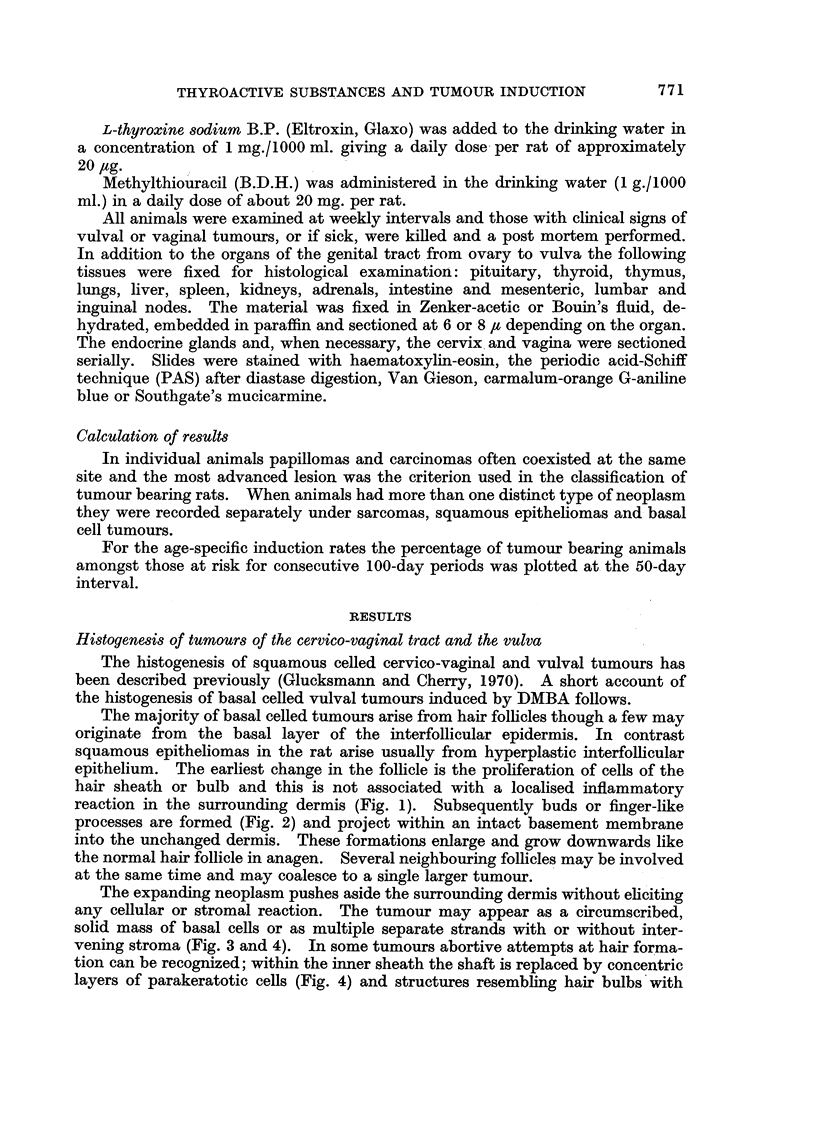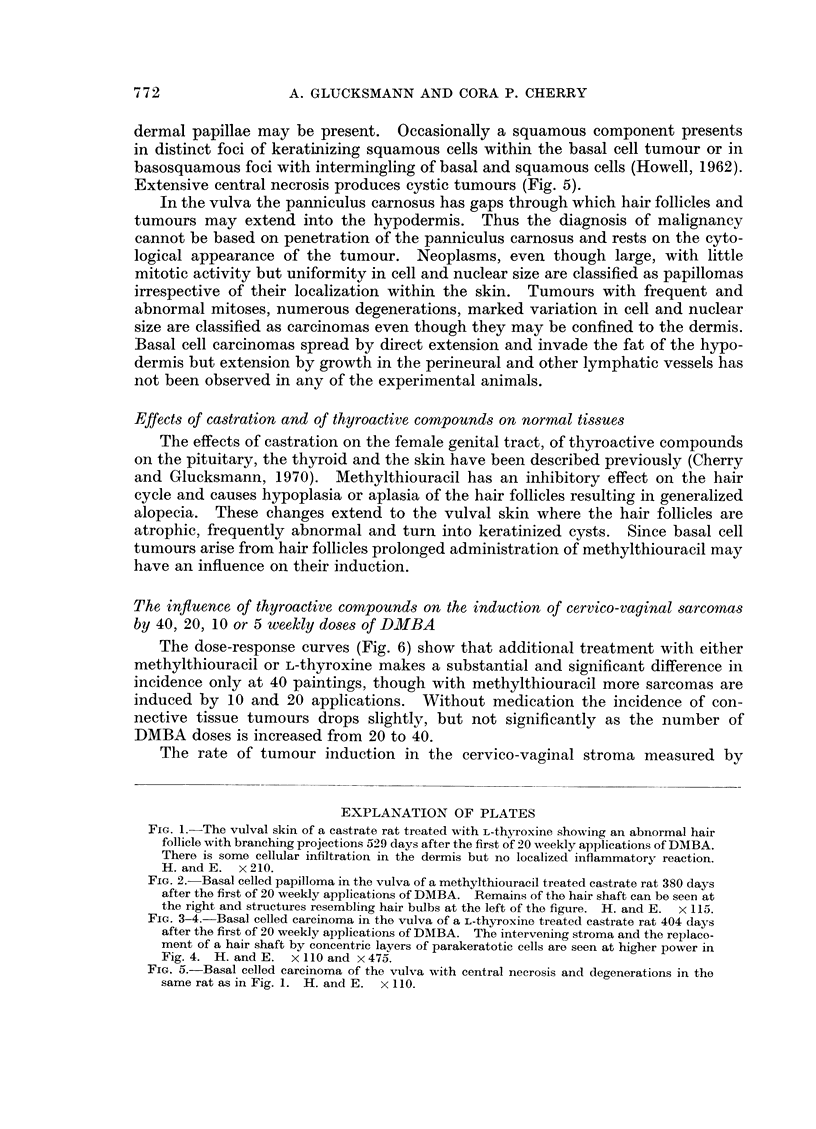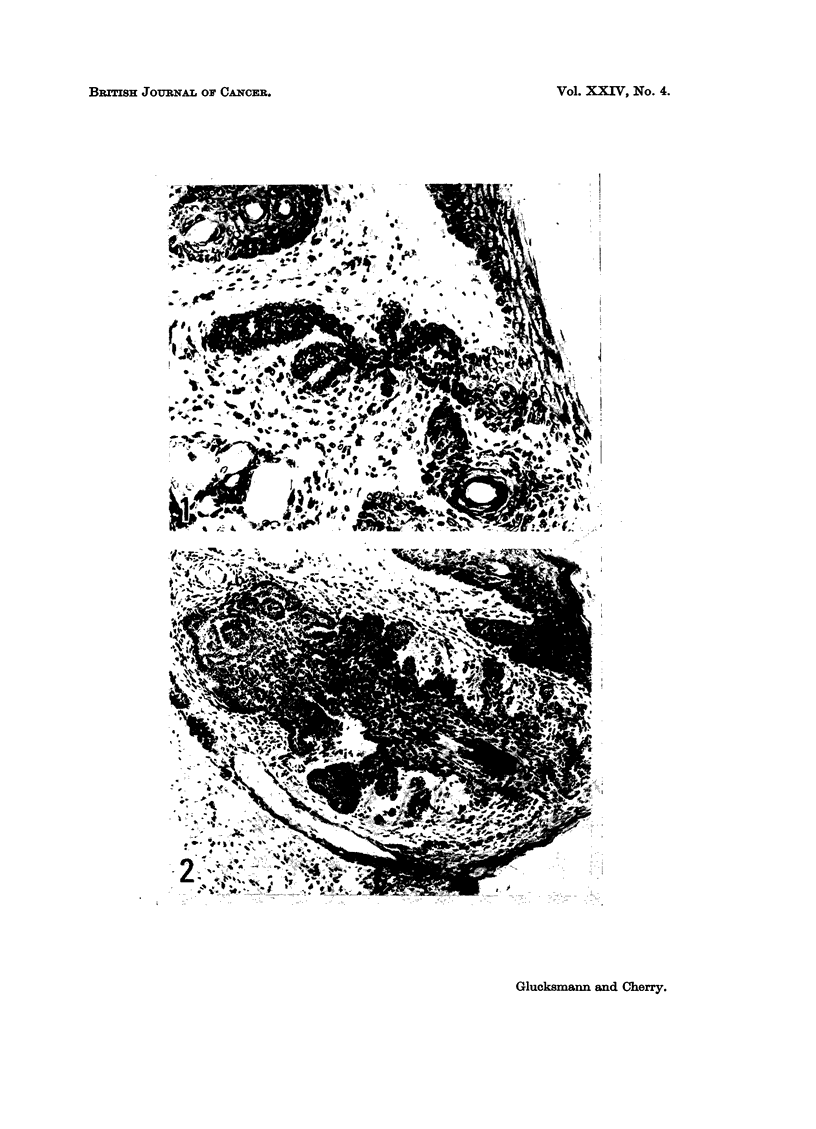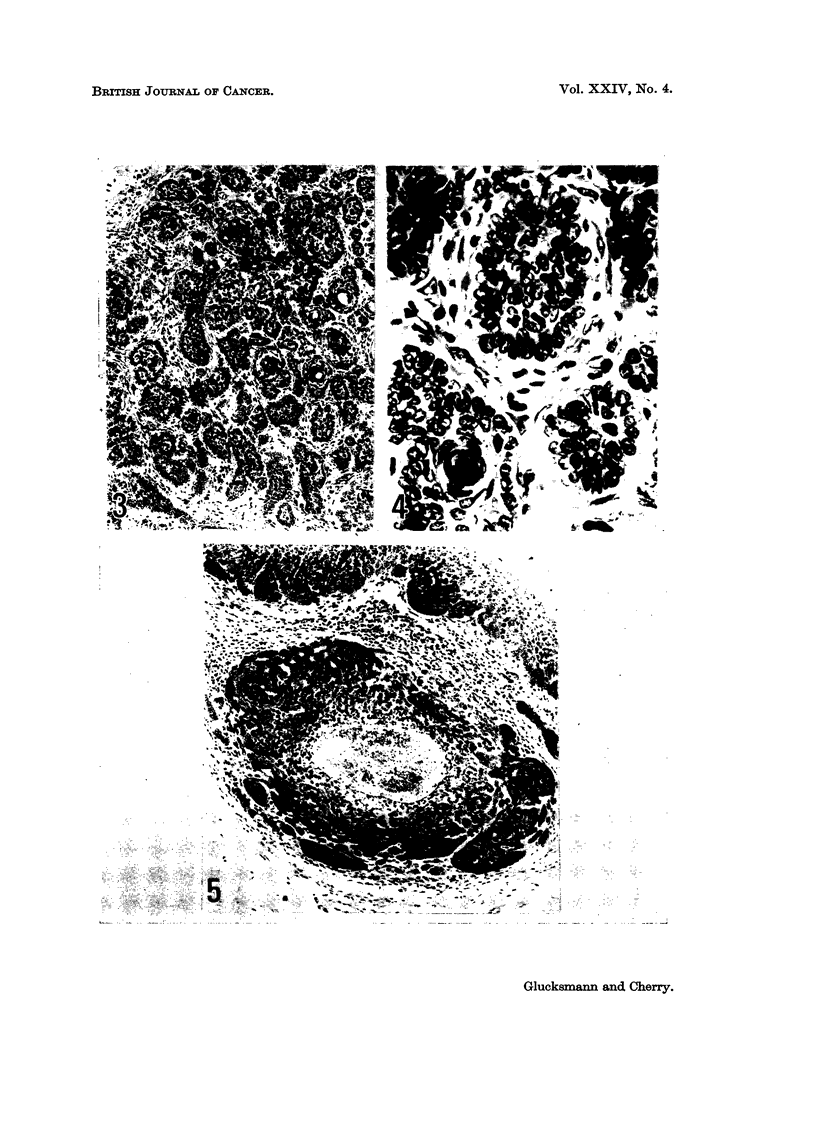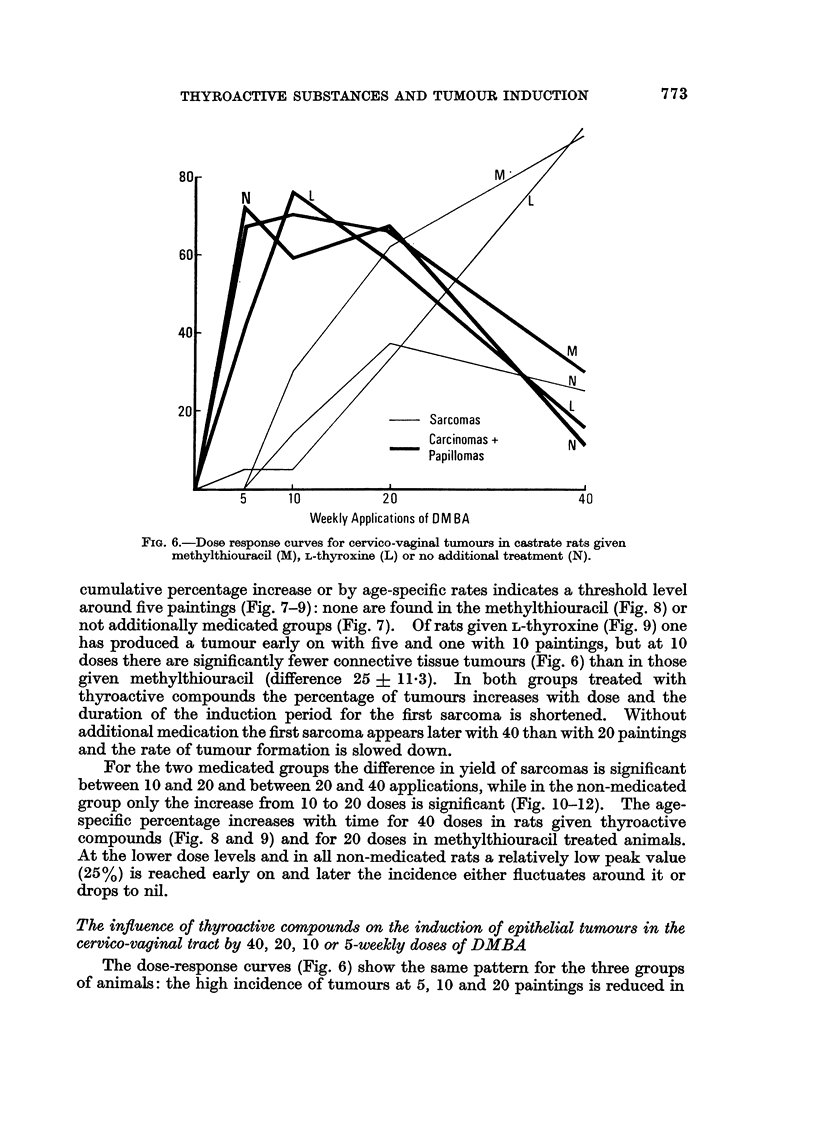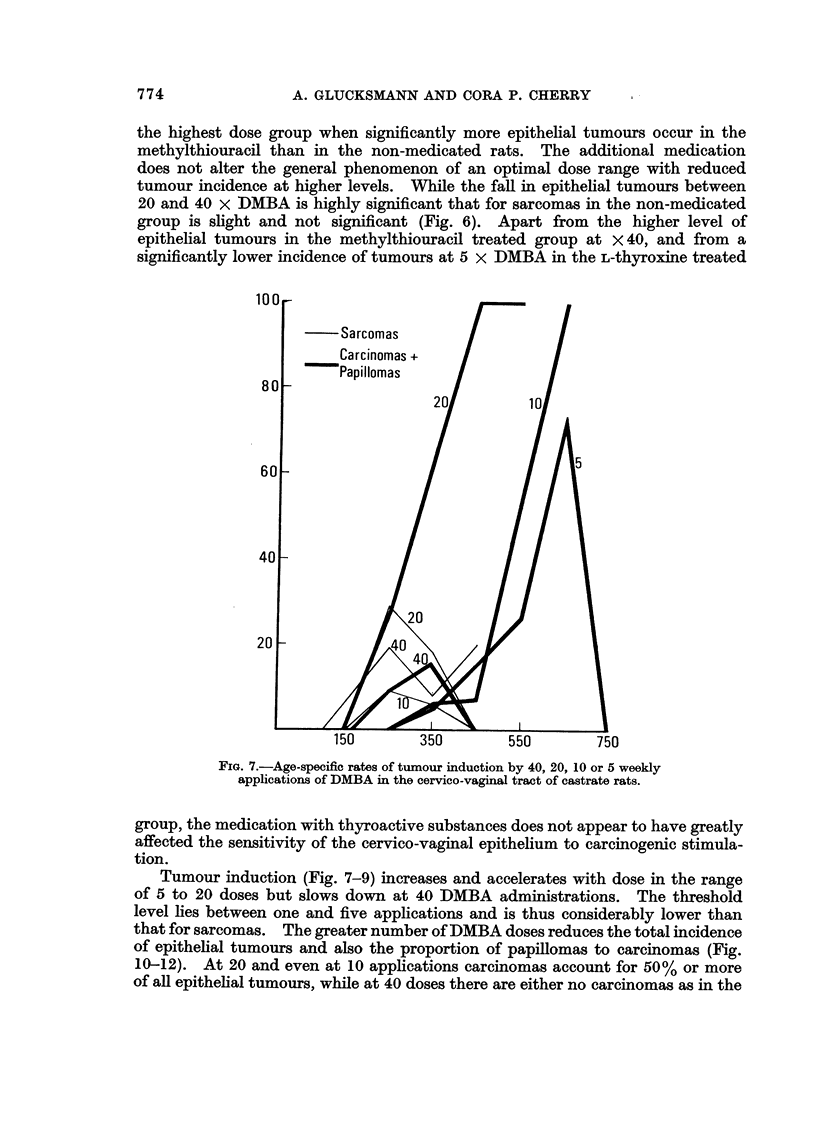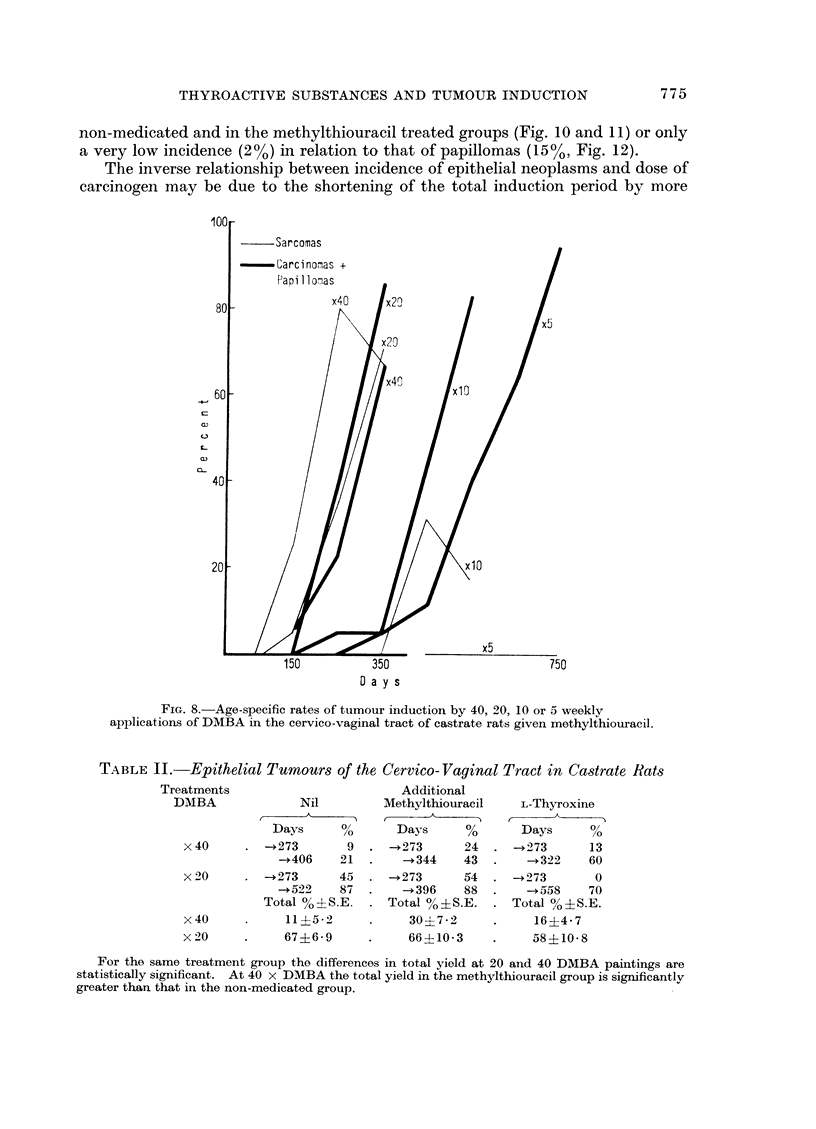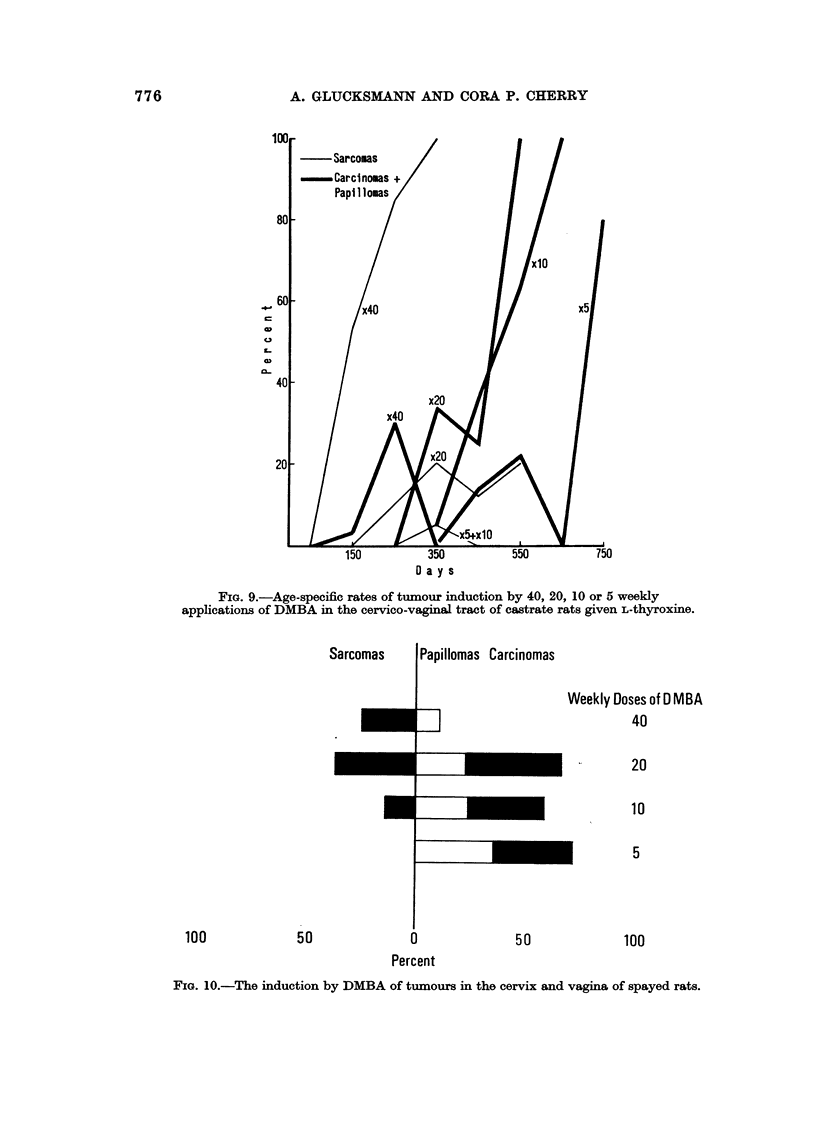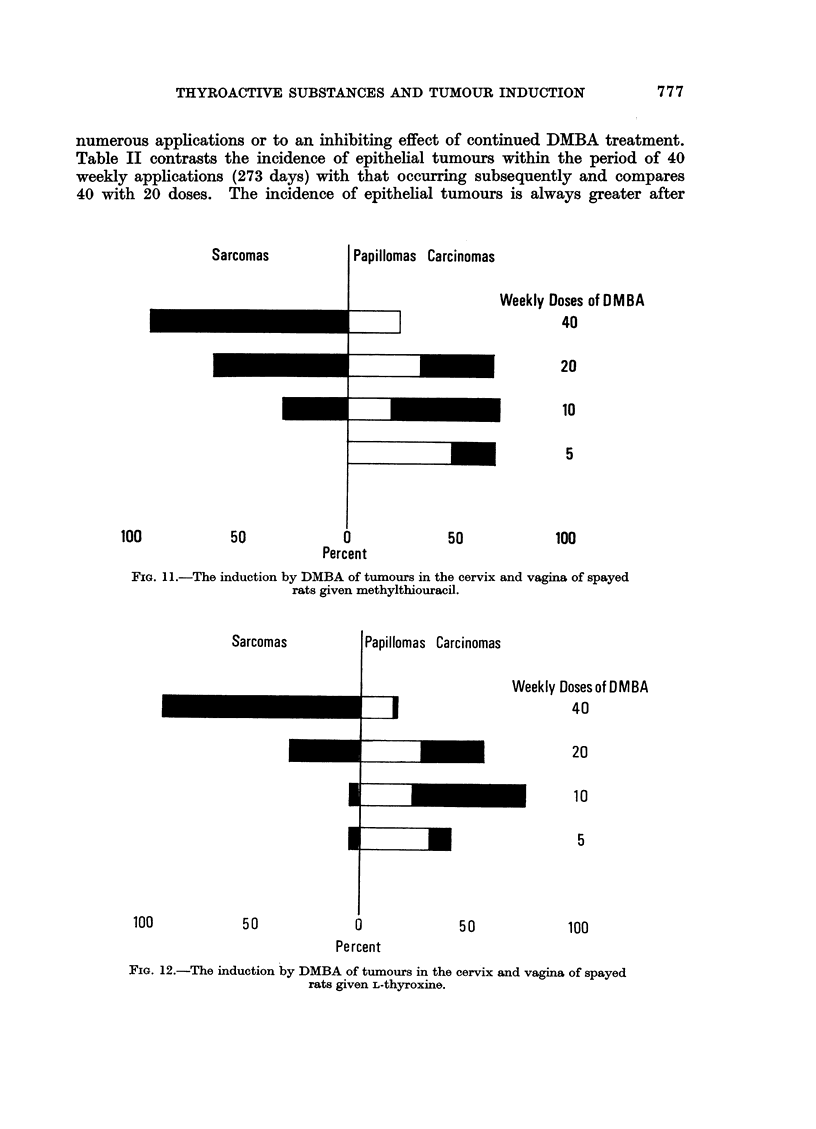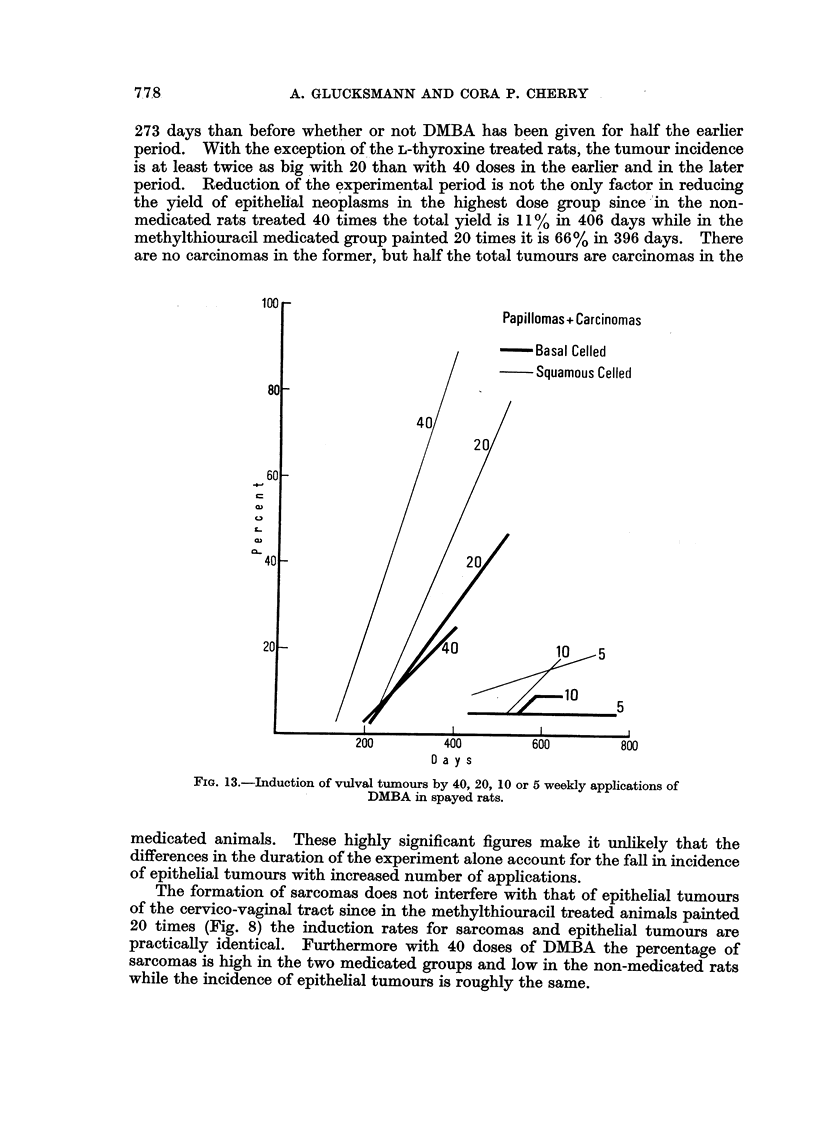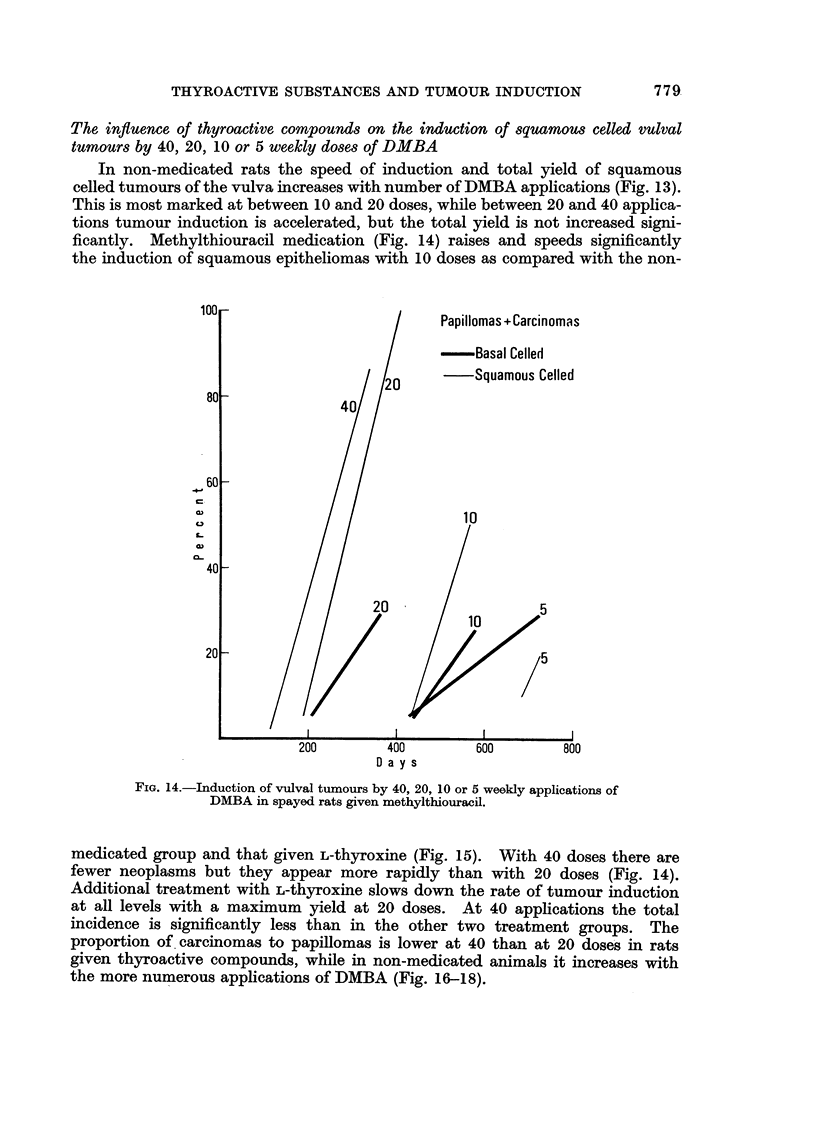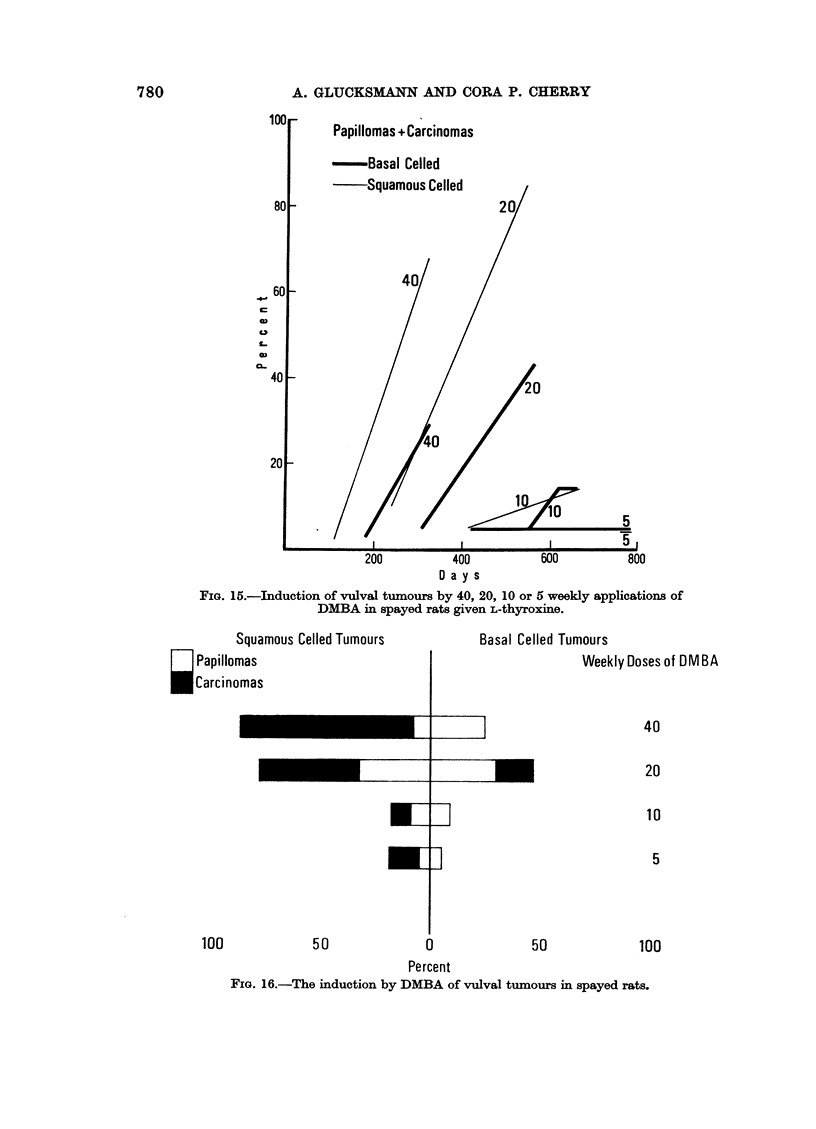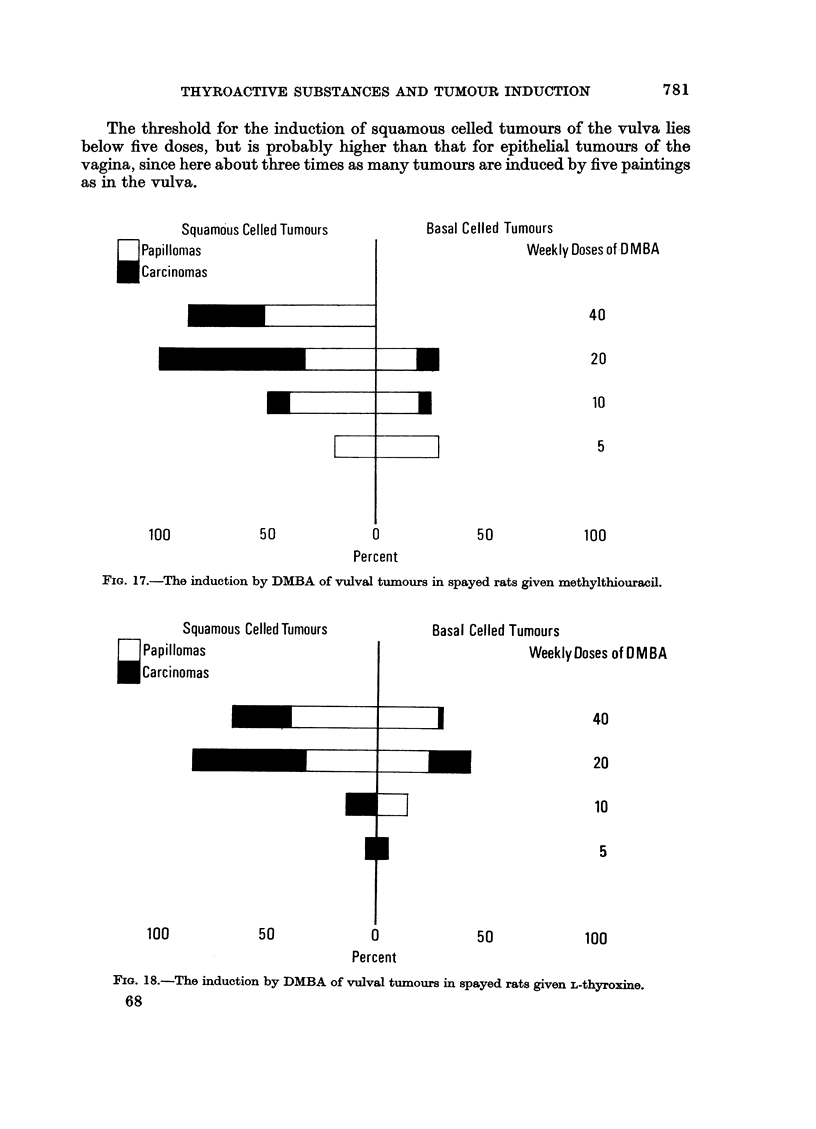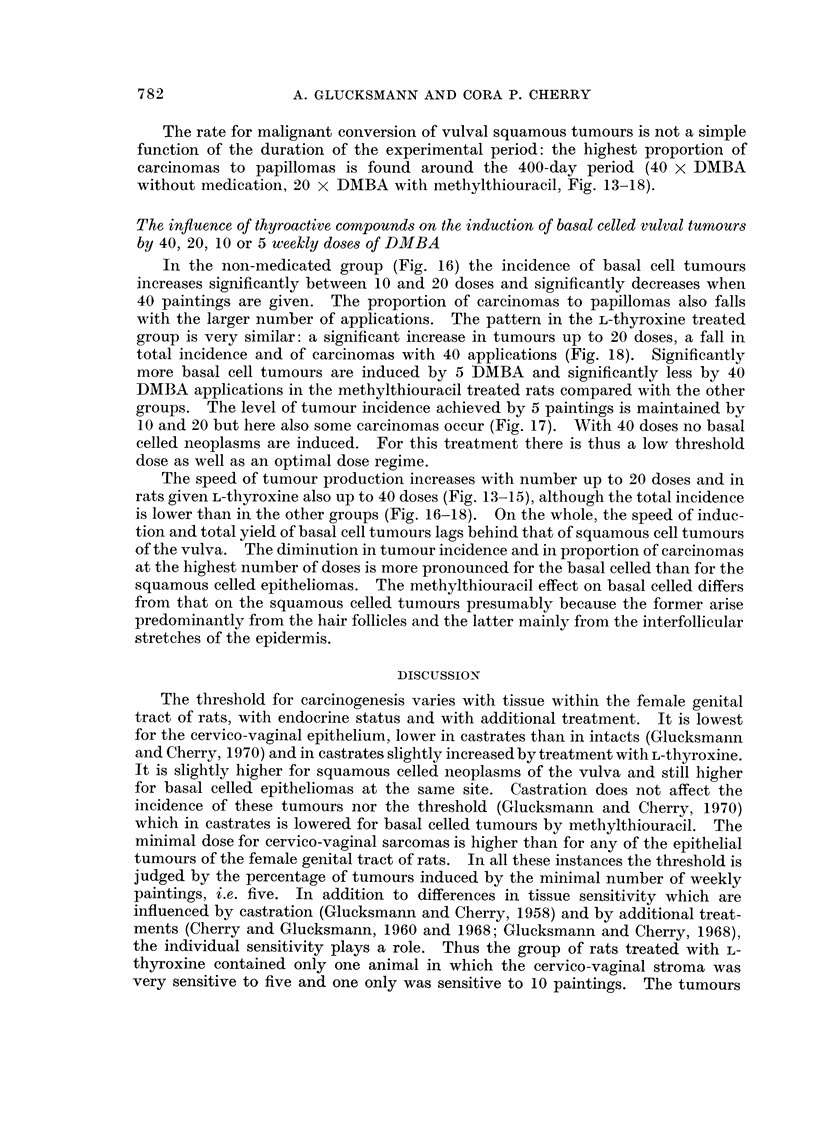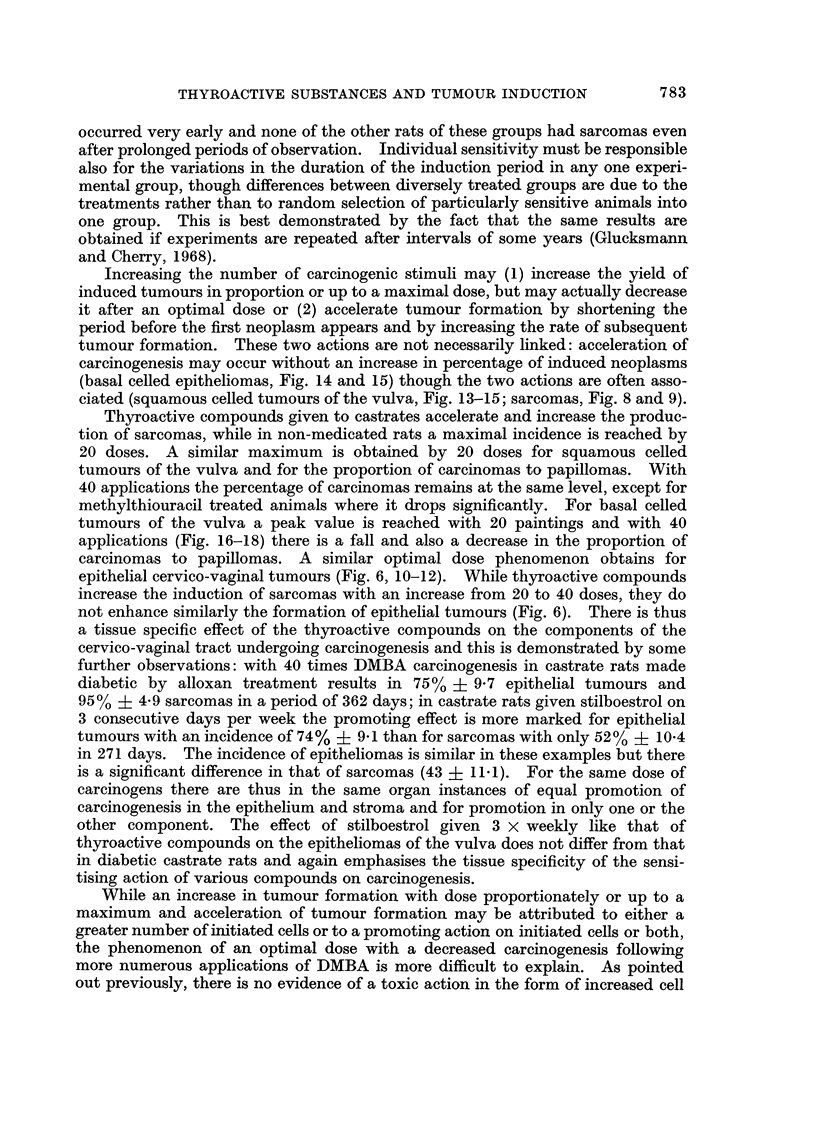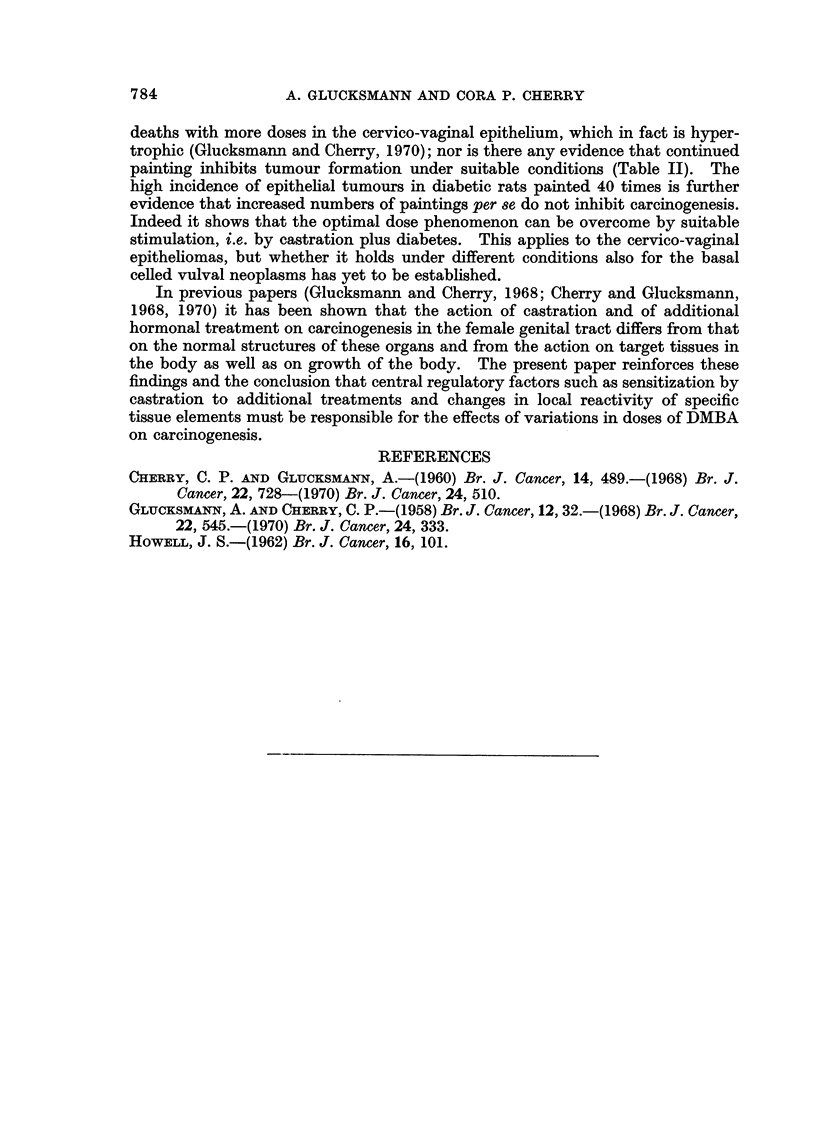# The Effect of Thyroactive Substances on the Induction of Cervico-vaginal and Vulval Tumours in Castrate Rats at Various Levels of Carcinogenic Treatment

**DOI:** 10.1038/bjc.1970.92

**Published:** 1970-12

**Authors:** A. Glucksmann, Cora P. Cherry

## Abstract

**Images:**


### THE EFFECT OF THYROACTIVE SUBSTANCES ON THE INDUCTION OF CERVICO-VAGINAL AND VULVAL TUMOURS IN CASTRATE RATS AT VARIOUS LEVELS OF CARCINOGENIC TREATMENT

A. GLUCKSMANN* AND CORA P. CHERRY†

*From the Strangeways Research Laboratory, Cambridge*

Received for publication July 31, 1970

**SUMMARY.—Medication with L-thyroxine or methylthiouracil of castrate rats painted weekly 5, 10, 20 or 40 times with DMBA does not alter the order of thresholds for carcinogenesis which increases from that for cervico-vaginal epitheliomas via squamous celled and basal celled vulval tumours to cervico-vaginal sarcomas. Methylthiouracil lowers the threshold for basal celled vulval neoplasms.**

**Sarcomas reach a peak of 25% with 20 doses of DMBA in non-medicated rats, but rise to 90% and at a faster rate in animals given either of the thyroactive drugs with further carcinogenic treatment.**

**The optimal dose phenomenon for cervico-vaginal epitheliomas, *i.e.* a significant fall with continued painting from a peak reached by 5 to 20 doses of DMBA, is not affected by medication with methylthiouracil or L-thyroxine.**

**Thyroactive compounds accelerate the formation of squamous celled vulval tumours which reach a maximum with 20 DMBA paintings; the total incidence as well as the proportion of carcinomas to papillomas falls with further treatment.**

**Methylthiouracil promotes formation of basal celled vulval tumours at low dose levels, but inhibits it at the highest. In medicated as in non-medicated rats the induction of basal celled tumours of the vulva follows an optimum dose pattern.**

**The optimal dose phenomenon and the effect of thyroactive compounds on the tissue-specific sensitivity to carcinogens are discussed.**

IN intact rats the incidence and rate of development of cervico-vaginal sarcomas increases with increasing numbers of weekly applications of DMBA ranging from 5 to 40, but in castrates a maximum incidence occurs at 20 doses and further applications fail to increase the yield of these neoplasms. If, however, at the level of about 40 doses castrate rats are given thyroactive substances such as methylthiouracil or L-thyroxine in the drinking water, the incidence of sarcomas is increased and accelerated and surpasses that in intact animals. More epithelial tumours of the cervico-vaginal tract are induced in castrate than in intact animals. The high incidence with 5 to 20 weekly applications drops significantly with 40 paintings, thus establishing clearly an optimal regime of carcinogenic stimulation. While in most instances increases in carcinogenic dosage cause either an increase

* Gibb Senior Fellow, Cancer Campaign for Research.
† Working with a grant from the Cancer Campaign for Research.

in neoplasms or maintain tumour incidence at a maximal level as in the case of cervico-vaginal sarcomas in castrates, the incidence of epithelial cervico-vaginal tumours definitely decreases with increasing dosage.

For a given dose more tumours can be elicited by additional treatment with various substances such as methylthiouracil or L-thyroxine. It thus seems possible that administration of these substances might also modify the optimal dosage phenomenon and thus shed some light on its causation and mechanism.

For the present experiments castrate rats have been used since the phenomena of optimal dosage, of maximal tumour incidence as well as of increase in tumour incidence with dosage are more clearly seen than in similarly treated intact animals. A comparison is made between castrate rats without additional treatment and those given additionally either L-thyroxine or methylthiouracil in the drinking water. All three treatment groups of animals have been painted with DMBA either 5, 10, 20 or 40 times weekly.

### MATERIALS AND METHODS

Hooded rats of the Lister strain, random bred within a closed colony in this laboratory since 1940, were used. The animals were housed not more than seven to a cage and given water and food pellets of MRC-diet 86 *ad libitum.* The thyroactive substances were dissolved and administered in the drinking water. Only animals surviving for at least 100 days after starting the experiment were considered at risk and the number of rats in the various treatment groups are given in Table I.

TABLE I.—*Additional Treatment, Number of Rats at Risk for Different Numbers of Weekly Applications of DMBA and Duration of Experiment*

| Additional treatment | Number at risk | DMBA | Duration of experiment (days) |
|---|---|---|---|
| None | 22 | ×5 | 770 |
| | 22 | ×10 | 647 |
| | 46 | ×20 | 522 |
| | 36 | ×40 | 406 |
| L-thyroxine | 19 | ×5 | 780 |
| | 21 | ×10 | 669 |
| | 21 | ×20 | 558 |
| | 61 | ×40 | 322 |
| Methylthiouracil | 21 | ×5 | 725 |
| | 20 | ×10 | 578 |
| | 21 | ×20 | 396 |
| | 40 | ×40 | 323 |

Bilateral ovariectomy was performed with a dorsal approach under ether anaesthesia on rats aged 5 to 6 weeks and carcinogenic treatment with a 1% solution in acetone of 9,10-dimethyl-1,2-benzanthracene (DMBA, Koch-Light, Ltd.) was started 2 to 3 weeks later. The vagina was stretched open by dorsal flexion of the tail and the solution was applied by means of a cotton wool swab mounted on a thin wire rod; it was distributed through a rotary motion over the cervix, vagina and introitus. This procedure was repeated at weekly intervals either for the life span of the animals (with an average of 40 applications) or restricted to 5, 10 or 20 times (Table I).

*L-thyroxine sodium* B.P. (Eltroxin, Glaxo) was added to the drinking water in a concentration of 1 mg./1000 ml. giving a daily dose per rat of approximately 20 $\mu$g.

Methylthiouracil (B.D.H.) was administered in the drinking water (1 g./1000 ml.) in a daily dose of about 20 mg. per rat.

All animals were examined at weekly intervals and those with clinical signs of vulval or vaginal tumours, or if sick, were killed and a post mortem performed. In addition to the organs of the genital tract from ovary to vulva the following tissues were fixed for histological examination: pituitary, thyroid, thymus, lungs, liver, spleen, kidneys, adrenals, intestine and mesenteric, lumbar and inguinal nodes. The material was fixed in Zenker-acetic or Bouin's fluid, dehydrated, embedded in paraffin and sectioned at 6 or 8 $\mu$ depending on the organ. The endocrine glands and, when necessary, the cervix and vagina were sectioned serially. Slides were stained with haematoxylin-eosin, the periodic acid-Schiff technique (PAS) after diastase digestion, Van Gieson, carmalum-orange G-aniline blue or Southgate's mucicarmine.

*Calculation of results*

In individual animals papillomas and carcinomas often coexisted at the same site and the most advanced lesion was the criterion used in the classification of tumour bearing rats. When animals had more than one distinct type of neoplasm they were recorded separately under sarcomas, squamous epitheliomas and basal cell tumours.

For the age-specific induction rates the percentage of tumour bearing animals amongst those at risk for consecutive 100-day periods was plotted at the 50-day interval.

### RESULTS

*Histogenesis of tumours of the cervico-vaginal tract and the vulva*

The histogenesis of squamous celled cervico-vaginal and vulval tumours has been described previously (Glucksmann and Cherry, 1970). A short account of the histogenesis of basal celled vulval tumours induced by DMBA follows.

The majority of basal celled tumours arise from hair follicles though a few may originate from the basal layer of the interfollicular epidermis. In contrast squamous epitheliomas in the rat arise usually from hyperplastic interfollicular epithelium. The earliest change in the follicle is the proliferation of cells of the hair sheath or bulb and this is not associated with a localised inflammatory reaction in the surrounding dermis (Fig. 1). Subsequently buds or finger-like processes are formed (Fig. 2) and project within an intact basement membrane into the unchanged dermis. These formations enlarge and grow downwards like the normal hair follicle in anagen. Several neighbouring follicles may be involved at the same time and may coalesce to a single larger tumour.

The expanding neoplasm pushes aside the surrounding dermis without eliciting any cellular or stromal reaction. The tumour may appear as a circumscribed, solid mass of basal cells or as multiple separate strands with or without intervening stroma (Fig. 3 and 4). In some tumours abortive attempts at hair formation can be recognized; within the inner sheath the shaft is replaced by concentric layers of parakeratotic cells (Fig. 4) and structures resembling hair bulbs with dermal papillae may be present. Occasionally a squamous component presents in distinct foci of keratinizing squamous cells within the basal cell tumour or in basosquamous foci with intermingling of basal and squamous cells (Howell, 1962). Extensive central necrosis produces cystic tumours (Fig. 5).

In the vulva the panniculus carnosus has gaps through which hair follicles and tumours may extend into the hypodermis. Thus the diagnosis of malignancy cannot be based on penetration of the panniculus carnosus and rests on the cytological appearance of the tumour. Neoplasms, even though large, with little mitotic activity but uniformity in cell and nuclear size are classified as papillomas irrespective of their localization within the skin. Tumours with frequent and abnormal mitoses, numerous degenerations, marked variation in cell and nuclear size are classified as carcinomas even though they may be confined to the dermis. Basal cell carcinomas spread by direct extension and invade the fat of the hypodermis but extension by growth in the perineural and other lymphatic vessels has not been observed in any of the experimental animals.

*Effects of castration and of thyroactive compounds on normal tissues*

The effects of castration on the female genital tract, of thyroactive compounds on the pituitary, the thyroid and the skin have been described previously (Cherry and Glucksmann, 1970). Methylthiouracil has an inhibitory effect on the hair cycle and causes hypoplasia or aplasia of the hair follicles resulting in generalized alopecia. These changes extend to the vulval skin where the hair follicles are atrophic, frequently abnormal and turn into keratinized cysts. Since basal cell tumours arise from hair follicles prolonged administration of methylthiouracil may have an influence on their induction.

*The influence of thyroactive compounds on the induction of cervico-vaginal sarcomas by* 40, 20, 10 *or* 5 *weekly doses of DMBA*

The dose-response curves (Fig. 6) show that additional treatment with either methylthiouracil or L-thyroxine makes a substantial and significant difference in incidence only at 40 paintings, though with methylthiouracil more sarcomas are induced by 10 and 20 applications. Without medication the incidence of connective tissue tumours drops slightly, but not significantly as the number of DMBA doses is increased from 20 to 40.

The rate of tumour induction in the cervico-vaginal stroma measured by

---

EXPLANATION OF PLATES

FIG. 1.—The vulval skin of a castrate rat treated with L-thyroxine showing an abnormal hair follicle with branching projections 529 days after the first of 20 weekly applications of DMBA. There is some cellular infiltration in the dermis but no localized inflammatory reaction. H. and E. ×210.

FIG. 2.—Basal celled papilloma in the vulva of a methylthiouracil treated castrate rat 380 days after the first of 20 weekly applications of DMBA. Remains of the hair shaft can be seen at the right and structures resembling hair bulbs at the left of the figure. H. and E. ×115.

FIG. 3–4.—Basal celled carcinoma in the vulva of a L-thyroxine treated castrate rat 404 days after the first of 20 weekly applications of DMBA. The intervening stroma and the replacement of a hair shaft by concentric layers of parakeratotic cells are seen at higher power in Fig. 4. H. and E. ×110 and ×475.

FIG. 5.—Basal celled carcinoma of the vulva with central necrosis and degenerations in the same rat as in Fig. 1. H. and E. ×110.

Glucksmann and Cherry.

3

4

5

FIG. 6.—Dose response curves for cervico-vaginal tumours in castrate rats given methylthiouracil (M), L-thyroxine (L) or no additional treatment (N).

cumulative percentage increase or by age-specific rates indicates a threshold level around five paintings (Fig. 7–9): none are found in the methylthiouracil (Fig. 8) or not additionally medicated groups (Fig. 7). Of rats given L-thyroxine (Fig. 9) one has produced a tumour early on with five and one with 10 paintings, but at 10 doses there are significantly fewer connective tissue tumours (Fig. 6) than in those given methylthiouracil (difference $25 \pm 11{\cdot}3$). In both groups treated with thyroactive compounds the percentage of tumours increases with dose and the duration of the induction period for the first sarcoma is shortened. Without additional medication the first sarcoma appears later with 40 than with 20 paintings and the rate of tumour formation is slowed down.

For the two medicated groups the difference in yield of sarcomas is significant between 10 and 20 and between 20 and 40 applications, while in the non-medicated group only the increase from 10 to 20 doses is significant (Fig. 10–12). The age-specific percentage increases with time for 40 doses in rats given thyroactive compounds (Fig. 8 and 9) and for 20 doses in methylthiouracil treated animals. At the lower dose levels and in all non-medicated rats a relatively low peak value (25%) is reached early on and later the incidence either fluctuates around it or drops to nil.

*The influence of thyroactive compounds on the induction of epithelial tumours in the cervico-vaginal tract by* 40, 20, 10 *or* 5*-weekly doses of DMBA*

The dose-response curves (Fig. 6) show the same pattern for the three groups of animals: the high incidence of tumours at 5, 10 and 20 paintings is reduced in the highest dose group when significantly more epithelial tumours occur in the methylthiouracil than in the non-medicated rats. The additional medication does not alter the general phenomenon of an optimal dose range with reduced tumour incidence at higher levels. While the fall in epithelial tumours between 20 and 40 × DMBA is highly significant that for sarcomas in the non-medicated group is slight and not significant (Fig. 6). Apart from the higher level of epithelial tumours in the methylthiouracil treated group at ×40, and from a significantly lower incidence of tumours at 5 × DMBA in the L-thyroxine treated

FIG. 7.—Age-specific rates of tumour induction by 40, 20, 10 or 5 weekly applications of DMBA in the cervico-vaginal tract of castrate rats.

group, the medication with thyroactive substances does not appear to have greatly affected the sensitivity of the cervico-vaginal epithelium to carcinogenic stimulation.

Tumour induction (Fig. 7–9) increases and accelerates with dose in the range of 5 to 20 doses but slows down at 40 DMBA administrations. The threshold level lies between one and five applications and is thus considerably lower than that for sarcomas. The greater number of DMBA doses reduces the total incidence of epithelial tumours and also the proportion of papillomas to carcinomas (Fig. 10–12). At 20 and even at 10 applications carcinomas account for 50% or more of all epithelial tumours, while at 40 doses there are either no carcinomas as in the non-medicated and in the methylthiouracil treated groups (Fig. 10 and 11) or only a very low incidence (2%) in relation to that of papillomas (15%, Fig. 12).

The inverse relationship between incidence of epithelial neoplasms and dose of carcinogen may be due to the shortening of the total induction period by more

FIG. 8.—Age-specific rates of tumour induction by 40, 20, 10 or 5 weekly applications of DMBA in the cervico-vaginal tract of castrate rats given methylthiouracil.

TABLE II.—*Epithelial Tumours of the Cervico-Vaginal Tract in Castrate Rats*

| Treatments DMBA | Nil | | Additional Methylthiouracil | | L-Thyroxine | |
|---|---|---|---|---|---|---|
| | Days | % | Days | % | Days | % |
| ×40 | →273 | 9 | →273 | 24 | →273 | 13 |
| | →406 | 21 | →344 | 43 | →322 | 60 |
| ×20 | →273 | 45 | →273 | 54 | →273 | 0 |
| | →522 | 87 | →396 | 88 | →558 | 70 |
| | Total %±S.E. | | Total %±S.E. | | Total %±S.E. | |
| ×40 | 11±5·2 | | 30±7·2 | | 16±4·7 | |
| ×20 | 67±6·9 | | 66±10·3 | | 58±10·8 | |

For the same treatment group the differences in total yield at 20 and 40 DMBA paintings are statistically significant. At 40 × DMBA the total yield in the methylthiouracil group is significantly greater than that in the non-medicated group.

FIG. 9.—Age-specific rates of tumour induction by 40, 20, 10 or 5 weekly applications of DMBA in the cervico-vaginal tract of castrate rats given L-thyroxine.

FIG. 10.—The induction by DMBA of tumours in the cervix and vagina of spayed rats.

numerous applications or to an inhibiting effect of continued DMBA treatment. Table II contrasts the incidence of epithelial tumours within the period of 40 weekly applications (273 days) with that occurring subsequently and compares 40 with 20 doses. The incidence of epithelial tumours is always greater after

Fig. 11.—The induction by DMBA of tumours in the cervix and vagina of spayed rats given methylthiouracil.

Fig. 12.—The induction by DMBA of tumours in the cervix and vagina of spayed rats given L-thyroxine.

273 days than before whether or not DMBA has been given for half the earlier period. With the exception of the L-thyroxine treated rats, the tumour incidence is at least twice as big with 20 than with 40 doses in the earlier and in the later period. Reduction of the experimental period is not the only factor in reducing the yield of epithelial neoplasms in the highest dose group since in the non-medicated rats treated 40 times the total yield is 11% in 406 days while in the methylthiouracil medicated group painted 20 times it is 66% in 396 days. There are no carcinomas in the former, but half the total tumours are carcinomas in the

FIG. 13.—Induction of vulval tumours by 40, 20, 10 or 5 weekly applications of DMBA in spayed rats.

medicated animals. These highly significant figures make it unlikely that the differences in the duration of the experiment alone account for the fall in incidence of epithelial tumours with increased number of applications.

The formation of sarcomas does not interfere with that of epithelial tumours of the cervico-vaginal tract since in the methylthiouracil treated animals painted 20 times (Fig. 8) the induction rates for sarcomas and epithelial tumours are practically identical. Furthermore with 40 doses of DMBA the percentage of sarcomas is high in the two medicated groups and low in the non-medicated rats while the incidence of epithelial tumours is roughly the same.

*The influence of thyroactive compounds on the induction of squamous celled vulval tumours by 40, 20, 10 or 5 weekly doses of DMBA*

In non-medicated rats the speed of induction and total yield of squamous celled tumours of the vulva increases with number of DMBA applications (Fig. 13). This is most marked at between 10 and 20 doses, while between 20 and 40 applications tumour induction is accelerated, but the total yield is not increased significantly. Methylthiouracil medication (Fig. 14) raises and speeds significantly the induction of squamous epitheliomas with 10 doses as compared with the non-

FIG. 14.—Induction of vulval tumours by 40, 20, 10 or 5 weekly applications of DMBA in spayed rats given methylthiouracil.

medicated group and that given L-thyroxine (Fig. 15). With 40 doses there are fewer neoplasms but they appear more rapidly than with 20 doses (Fig. 14). Additional treatment with L-thyroxine slows down the rate of tumour induction at all levels with a maximum yield at 20 doses. At 40 applications the total incidence is significantly less than in the other two treatment groups. The proportion of carcinomas to papillomas is lower at 40 than at 20 doses in rats given thyroactive compounds, while in non-medicated animals it increases with the more numerous applications of DMBA (Fig. 16–18).

FIG. 15.—Induction of vulval tumours by 40, 20, 10 or 5 weekly applications of DMBA in spayed rats given L-thyroxine.

FIG. 16.—The induction by DMBA of vulval tumours in spayed rats.

The threshold for the induction of squamous celled tumours of the vulva lies below five doses, but is probably higher than that for epithelial tumours of the vagina, since here about three times as many tumours are induced by five paintings as in the vulva.

FIG. 17.—The induction by DMBA of vulval tumours in spayed rats given methylthiouracil.

FIG. 18.—The induction by DMBA of vulval tumours in spayed rats given L-thyroxine.

The rate for malignant conversion of vulval squamous tumours is not a simple function of the duration of the experimental period: the highest proportion of carcinomas to papillomas is found around the 400-day period (40 × DMBA without medication, 20 × DMBA with methylthiouracil, Fig. 13–18).

*The influence of thyroactive compounds on the induction of basal celled vulval tumours by* 40, 20, 10 *or* 5 *weekly doses of DMBA*

In the non-medicated group (Fig. 16) the incidence of basal cell tumours increases significantly between 10 and 20 doses and significantly decreases when 40 paintings are given. The proportion of carcinomas to papillomas also falls with the larger number of applications. The pattern in the L-thyroxine treated group is very similar: a significant increase in tumours up to 20 doses, a fall in total incidence and of carcinomas with 40 applications (Fig. 18). Significantly more basal cell tumours are induced by 5 DMBA and significantly less by 40 DMBA applications in the methylthiouracil treated rats compared with the other groups. The level of tumour incidence achieved by 5 paintings is maintained by 10 and 20 but here also some carcinomas occur (Fig. 17). With 40 doses no basal celled neoplasms are induced. For this treatment there is thus a low threshold dose as well as an optimal dose regime.

The speed of tumour production increases with number up to 20 doses and in rats given L-thyroxine also up to 40 doses (Fig. 13–15), although the total incidence is lower than in the other groups (Fig. 16–18). On the whole, the speed of induction and total yield of basal cell tumours lags behind that of squamous cell tumours of the vulva. The diminution in tumour incidence and in proportion of carcinomas at the highest number of doses is more pronounced for the basal celled than for the squamous celled epitheliomas. The methylthiouracil effect on basal celled differs from that on the squamous celled tumours presumably because the former arise predominantly from the hair follicles and the latter mainly from the interfollicular stretches of the epidermis.

### DISCUSSION

The threshold for carcinogenesis varies with tissue within the female genital tract of rats, with endocrine status and with additional treatment. It is lowest for the cervico-vaginal epithelium, lower in castrates than in intacts (Glucksmann and Cherry, 1970) and in castrates slightly increased by treatment with L-thyroxine. It is slightly higher for squamous celled neoplasms of the vulva and still higher for basal celled epitheliomas at the same site. Castration does not affect the incidence of these tumours nor the threshold (Glucksmann and Cherry, 1970) which in castrates is lowered for basal celled tumours by methylthiouracil. The minimal dose for cervico-vaginal sarcomas is higher than for any of the epithelial tumours of the female genital tract of rats. In all these instances the threshold is judged by the percentage of tumours induced by the minimal number of weekly paintings, *i.e.* five. In addition to differences in tissue sensitivity which are influenced by castration (Glucksmann and Cherry, 1958) and by additional treatments (Cherry and Glucksmann, 1960 and 1968; Glucksmann and Cherry, 1968), the individual sensitivity plays a role. Thus the group of rats treated with L-thyroxine contained only one animal in which the cervico-vaginal stroma was very sensitive to five and one only was sensitive to 10 paintings. The tumours occurred very early and none of the other rats of these groups had sarcomas even after prolonged periods of observation. Individual sensitivity must be responsible also for the variations in the duration of the induction period in any one experimental group, though differences between diversely treated groups are due to the treatments rather than to random selection of particularly sensitive animals into one group. This is best demonstrated by the fact that the same results are obtained if experiments are repeated after intervals of some years (Glucksmann and Cherry, 1968).

Increasing the number of carcinogenic stimuli may (1) increase the yield of induced tumours in proportion or up to a maximal dose, but may actually decrease it after an optimal dose or (2) accelerate tumour formation by shortening the period before the first neoplasm appears and by increasing the rate of subsequent tumour formation. These two actions are not necessarily linked: acceleration of carcinogenesis may occur without an increase in percentage of induced neoplasms (basal celled epitheliomas, Fig. 14 and 15) though the two actions are often associated (squamous celled tumours of the vulva, Fig. 13–15; sarcomas, Fig. 8 and 9).

Thyroactive compounds given to castrates accelerate and increase the production of sarcomas, while in non-medicated rats a maximal incidence is reached by 20 doses. A similar maximum is obtained by 20 doses for squamous celled tumours of the vulva and for the proportion of carcinomas to papillomas. With 40 applications the percentage of carcinomas remains at the same level, except for methylthiouracil treated animals where it drops significantly. For basal celled tumours of the vulva a peak value is reached with 20 paintings and with 40 applications (Fig. 16–18) there is a fall and also a decrease in the proportion of carcinomas to papillomas. A similar optimal dose phenomenon obtains for epithelial cervico-vaginal tumours (Fig. 6, 10–12). While thyroactive compounds increase the induction of sarcomas with an increase from 20 to 40 doses, they do not enhance similarly the formation of epithelial tumours (Fig. 6). There is thus a tissue specific effect of the thyroactive compounds on the components of the cervico-vaginal tract undergoing carcinogenesis and this is demonstrated by some further observations: with 40 times DMBA carcinogenesis in castrate rats made diabetic by alloxan treatment results in $75\% \pm 9{\cdot}7$ epithelial tumours and $95\% \pm 4{\cdot}9$ sarcomas in a period of 362 days; in castrate rats given stilboestrol on 3 consecutive days per week the promoting effect is more marked for epithelial tumours with an incidence of $74\% \pm 9{\cdot}1$ than for sarcomas with only $52\% \pm 10{\cdot}4$ in 271 days. The incidence of epitheliomas is similar in these examples but there is a significant difference in that of sarcomas ($43 \pm 11{\cdot}1$). For the same dose of carcinogens there are thus in the same organ instances of equal promotion of carcinogenesis in the epithelium and stroma and for promotion in only one or the other component. The effect of stilboestrol given $3 \times$ weekly like that of thyroactive compounds on the epitheliomas of the vulva does not differ from that in diabetic castrate rats and again emphasises the tissue specificity of the sensitising action of various compounds on carcinogenesis.

While an increase in tumour formation with dose proportionately or up to a maximum and acceleration of tumour formation may be attributed to either a greater number of initiated cells or to a promoting action on initiated cells or both, the phenomenon of an optimal dose with a decreased carcinogenesis following more numerous applications of DMBA is more difficult to explain. As pointed out previously, there is no evidence of a toxic action in the form of increased cell deaths with more doses in the cervico-vaginal epithelium, which in fact is hypertrophic (Glucksmann and Cherry, 1970); nor is there any evidence that continued painting inhibits tumour formation under suitable conditions (Table II). The high incidence of epithelial tumours in diabetic rats painted 40 times is further evidence that increased numbers of paintings *per se* do not inhibit carcinogenesis. Indeed it shows that the optimal dose phenomenon can be overcome by suitable stimulation, *i.e.* by castration plus diabetes. This applies to the cervico-vaginal epitheliomas, but whether it holds under different conditions also for the basal celled vulval neoplasms has yet to be established.

In previous papers (Glucksmann and Cherry, 1968; Cherry and Glucksmann, 1968, 1970) it has been shown that the action of castration and of additional hormonal treatment on carcinogenesis in the female genital tract differs from that on the normal structures of these organs and from the action on target tissues in the body as well as on growth of the body. The present paper reinforces these findings and the conclusion that central regulatory factors such as sensitization by castration to additional treatments and changes in local reactivity of specific tissue elements must be responsible for the effects of variations in doses of DMBA on carcinogenesis.

### REFERENCES

Cherry, C. P. and Glucksmann, A.—(1960) *Br. J. Cancer*, **14**, 489.—(1968) *Br. J. Cancer*, **22**, 728—(1970) *Br. J. Cancer*, **24**, 510.

Glucksmann, A. and Cherry, C. P.—(1958) *Br. J. Cancer*, **12**, 32.—(1968) *Br. J. Cancer*, **22**, 545.—(1970) *Br. J. Cancer*, **24**, 333.

Howell, J. S.—(1962) *Br. J. Cancer*, **16**, 101.